# Speckle‐Based Optical Cryptosystem and its Application for Human Face Recognition via Deep Learning

**DOI:** 10.1002/advs.202202407

**Published:** 2022-06-24

**Authors:** Qi Zhao, Huanhao Li, Zhipeng Yu, Chi Man Woo, Tianting Zhong, Shengfu Cheng, Yuanjin Zheng, Honglin Liu, Jie Tian, Puxiang Lai

**Affiliations:** ^1^ Department of Biomedical Engineering Hong Kong Polytechnic University Hong Kong SAR; ^2^ Shenzhen Research Institute Hong Kong Polytechnic University Shenzhen 518057 China; ^3^ School of Electrical and Electronic Engineering Nanyang Technological University Singapore 639798 Singapore; ^4^ Key Laboratory for Quantum Optics, Shanghai Institute of Optics and Fine Mechanics Chinese Academy of Sciences Shanghai 201800 China; ^5^ Beijing Advanced Innovation Center for Big Data‐Based Precision Medicine, School of Medical Science and Engineering Beihang University Beijing 100191 China; ^6^ Key Laboratory of Molecular Imaging, Institute of Automation Chinese Academy of Sciences Beijing 100190 China; ^7^ Photonics Research Institute Hong Kong Polytechnic University Hong Kong SAR

**Keywords:** deep learning, face recognition, optical encryption, speckle

## Abstract

Face recognition has become ubiquitous for authentication or security purposes. Meanwhile, there are increasing concerns about the privacy of face images, which are sensitive biometric data and should be protected. Software‐based cryptosystems are widely adopted to encrypt face images, but the security level is limited by insufficient digital secret key length or computing power. Hardware‐based optical cryptosystems can generate enormously longer secret keys and enable encryption at light speed, but most reported optical methods, such as double random phase encryption, are less compatible with other systems due to system complexity. In this study, a plain yet highly efficient speckle‐based optical cryptosystem is proposed and implemented. A scattering ground glass is exploited to generate physical secret keys of 17.2 gigabit length and encrypt face images via seemingly random optical speckles at light speed. Face images can then be decrypted from random speckles by a well‐trained decryption neural network, such that face recognition can be realized with up to 98% accuracy. Furthermore, attack analyses are carried out to show the cryptosystem's security. Due to its high security, fast speed, and low cost, the speckle‐based optical cryptosystem is suitable for practical applications and can inspire other high‐security cryptosystems.

## Graphical abstract

A plain yet highly efficient speckle‐based optical cryptosystem is proposed, where a ground glass is exploited as the physical secret key with a 17.2 gigabit length to encrypt face images via random optical speckles at light speed. A well‐trained neural network can decrypt speckles to face images with high fidelity, such that face recognition accuracy can be up to 98%.

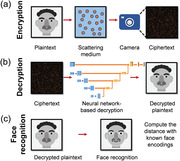

## 1 Introduction

The human face is a personal identifier, and an adult can hardly change the appearance. In modern society, numerous face recognition scenes have been set up for authentication or security purposes due to the increasing concern for personal privacy and public safety.^[^ ^1^ ^]^ The storage of human face data is hence highly confidential. If the face database is leaked, hackers may use this information to attack key sectors, including bank accounts.^[^ ^2^ ^]^ Therefore, effective protection of face image data is essential for privacy and security.^[^ ^3^ ^]^

Various cryptosystems, including software‐based and hardware‐based, have been put forward to protect private data. For software‐based cryptosystems, well‐known encryption algorithms have been developed, such as Rivest–Shamir–Adleman encryption (RSA),^[^ ^4^ ^]^ Advanced Encryption Standards (AES),^[^ ^5^ ^]^ Message Digest Algorithm (MD5),^[^ ^6^ ^]^ etc. These algorithms are all based on mathematical theories whose digital secret key lengths range from tens to hundreds of bits. The selection of the secret key lengths involves a trade‐off or balance between security level and processing speed. Such a limited key length seems to be sufficiently secure for conventional attacks by general computers but is vulnerable to attacks by the rapidly evolving quantum computers, whose computing power is 10^8^ times that of the general ones.^[^ ^7^ ^]^ As a result, researchers keep exploiting novel cryptosystems to achieve higher security, and hardware‐based solutions are therefore in demand.

Amongst current hardware‐based solutions, optical cryptosystems are of extensive interest with the development of optical computing and computational imaging.^[^ ^8^, ^9^ ^]^ The optical methods may lead to breakthroughs in cryptosystems due to their superior performance, such as fast speed, high security, low cost, etc.^[^ ^10^ ^]^ Generally, optical cryptosystems use diffracted light to obtain the ciphertext from the plaintext (data or images to be encrypted), thus there is no computational cost and high‐speed encryption (i.e., speed of light) is guaranteed. Moreover, the large dimensionality of the optical diffraction mechanism guarantees a long length for digital secret keys, resulting in higher security.^[^ ^11^ ^]^ In contrast, to achieve comparable secret key length in software‐based cryptosystems, a high‐performance computer is inevitable, and the cost is demanding. In view of these advantages, researchers have devised various optical cryptosystems, such as double random phase encryption (DRPE)^[^ ^12^, ^13^ ^]^ and speckle‐based optical cryptosystems.^[^ ^14^, ^15^ ^]^ DRPE uses two phase masks at the input plane and the plaintexts are then encrypted on the Fourier plane. Although DRPE has been investigated for more than two decades, it is not yet widely adopted as the optical design is complicated to be integrated with other systems. A most recent nonlinear optics‐based encryption study reported in early 2022 faces such a limitation for extensions due to its interferometric configuration.^[^ ^16^ ^]^

Speckle‐based approaches are therefore of interest, in which optical speckles are utilized as ciphertext to encrypt plaintext. Compared with DRPE, this method is much easier to implement with a plain optical setup. In a strong scattering regime, the plaintext (e.g., images) is optically scrambled, resulting in speckles featured by randomly distributed bright and dark regions, which can be captured by regular digital cameras for further processing. The random feature of the speckles seems meaningless and usually annoying, but constitutes nearly infinite information channels^[^ ^17^ ^]^ and hence the tremendously long physical secret key length in a cryptosystem,^[^ ^12^ ^]^ which can be exploited to yield high‐level security and information protection. Thus far, a few methods, such as based on transmission matrix,^[^ ^17^, ^18^ ^]^ support vector regression,^[^ ^19^ ^]^ neural networks,^[^ ^14^ ^]^ etc., have been developed to reconstruct images from the speckles. Among these approaches, neural networks can automatically learn the complex relationships between the plaintext and the ciphertext, resulting in image reconstruction of higher fidelity than other methods can yield.^[^ ^20^, ^21^, ^22^, ^23^, ^24^, ^25^ ^]^ Since the physical models in speckle‐based optical cryptosystems are similar to those for imaging through scattering media, neural networks can also be applied in speckle‐based optical cryptosystems to decrypt speckles for higher‐level applications like face recognition.

**Figure 1:**
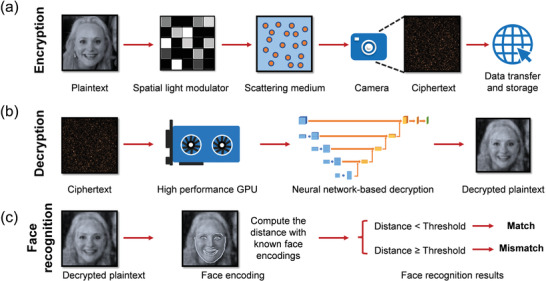
The flowchart of the proposed cryptosystem for face recognition. a) Speckle encryption: face images (plaintext) are loaded on a spatial light modulator (SLM) to generate the corresponding speckles (ciphertext) when coherent light reflected by the SLM transmits through a scattering medium, which serves as the unique physical secret key. The ciphertext is safely transferred and stored via the cloud. No face images need to be kept in the database after encryption. b) Learning‐based decryption: a neural network is trained in advance to link the plaintext with the ciphertext. After training, new random speckle patterns (ciphertext) are directly fed into the neural network for decryption, and the decrypted face images are then utilized for face recognition. c) Face recognition: the camera‐recorded face images are encoded to unique 128‐dimensional vectors of each known face image. After decryption, the face encoding distances between the decrypted images and the known face encodings are computed: if the encoding distance is less than a pre‐set threshold, the face recognition result is “Match” (the same person), otherwise it is “Mismatch” (different people). Plaintext image: Reproduced under terms of the CC‐BY 2.0 license. Copyright 2015, Lawrence Lessig at Second Home London, by Innotech Summit, Flickr (https://www.flickr.com/photos/115363358@N03/18260388752/). The original image is cropped and converted to gray‐scale.

It must be clarified that optical cryptosystems with high‐security and fast‐speed encryption have been investigated, and various applications in encrypting simple structural images (e.g., characters, clothes, animals, etc.) have been demonstrated.^[^ ^12^, ^13^, ^14^, ^15^, ^17^, ^26^ ^]^ However, speckle‐based optical cryptosystems for complex tasks, such as encrypted face recognition, have rarely been explored. The main challenge here is to decrypt images from rapidly changing optical speckles and to recognize faces from the decrypted images. Moreover, to achieve high accuracy in face recognition, decryption with high fidelity in key features and detailed structures is required. In this work, we propose a scheme that utilizes optical speckles for face image encryption and a deep neural network for speckle decryption, and the decrypted images are then used for face recognition. The concept, as illustrated in **Figure** **1**, can be decomposed into three stages: first, face images are optically scrambled into speckles for encryption, which protects the data during transmission and storage; then, a neural network is trained to decrypt the face images with high fidelity from the ciphertext (i.e., speckles); last, the decrypted images are compared with the known face encodings and recognized. In this cryptosystem, face images are encrypted into seemingly random speckles that are nearly impossible to be decrypted without the knowledge of the physical key (i.e., the scattering medium) or the learned digital key (i.e., the trained neural network). Moreover, only speckles but no face images are stored in the database to avoid any potential private information leakage. To the best of our knowledge, this is the first demonstration of a speckle‐based optical cryptosystem for face recognition, and the accuracy in this study has reached more than 98%, which is applicable in a wide range of applications.

## 2 Results

### 2.1 Speckle‐Based Encryption

**Figure 2:**
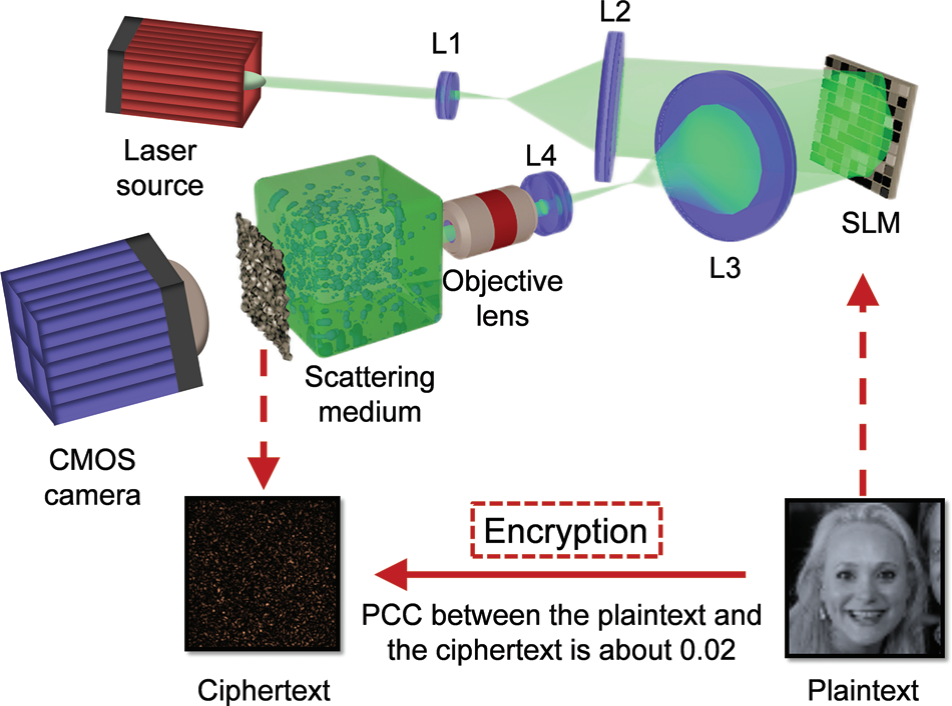
The optical setup for encryption. Face images (plaintext) are displayed on the SLM, which is illuminated by an expanded continuous coherent laser beam (*λ* = 532 nm), generating speckles (ciphertext) through a scattering medium. The speckles are recorded by a complementary metal‐oxide‐semiconductor (CMOS) camera, which is synchronized by a Matlab program to ensure one‐to‐one mapping with the displayed face image for network training. Plaintext image: Reproduced under terms of the CC‐BY 2.0 license. Copyright 2015, Lawrence Lessig at Second Home London, by Innotech Summit, Flickr (https://www.flickr.com/photos/115363358@N03/18260388752/). The original image is cropped and converted to gray‐scale.

**Figure** **2** shows the experimental optical setup for information encryption (see Experimental Section for details). Face images from the “Flickr Faces High Quality” (FFHQ) database^[^ ^27^ ^]^ are displayed on a phase‐modulating spatial light modulator (SLM) to modulate the incident coherent light from a 532 nm single mode laser source (300 mW, EXLSR‐532‐300‐CDRH, Spectra‐Physics, USA). Thus, the information of the face images (i.e., plaintext) is carried by the wavefront modulated laser beam. Then, the modulated wavefront passes through a scattering medium (220‐grid ground glass, DG10‐220‐MD, Thorlabs, USA) and is multiply scattered to form random speckles (i.e., ciphertext), which are captured by a digital camera (FL3‐U3‐32S2M‐CS, PointGrey, Canada). During encryption, which is the process of generating speckles, a MATLAB program synchronizes all devices to ensure each captured speckle pattern (i.e., ciphertext) is paired with one exclusive face image (i.e., plaintext) displayed on the SLM, as illustrated in Figure 2. As seen, the ciphertext appears random and exhibits no direct relationship with the plaintext, and the mean Pearson correlation coefficient (PCC) between them is as low as 0.02.

### 2.2 Learning‐Based Decryption

**Figure 3:**
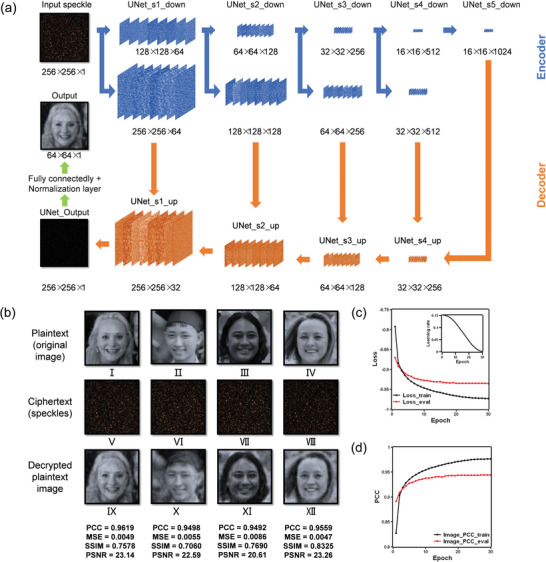
Neural network structure and the decryption performance. a) Architectures of the neural network based on U‐Net with an additional layer of a complex fully connected layer and normalization layer. The U‐Net mainly contains 4 layers, with 4 down‐sampling blocks for encoders (marked in blue) and 4 up‐sampling blocks for decoders (marked in orange).^[^ ^28^ ^]^ The final outputs are face images decrypted from speckles, which are then used for face recognition. The dimensions of the filters are described as length × height × amount, and the filters shown here are visualized by inputting one speckle pattern into the neural network. b) Four groups of exampled plaintexts, ciphertexts, and decrypted plaintext images during network testing. The ciphertexts are all from the same scattering medium, and the decrypted plaintext images are the results of inputting ciphertexts to the pre‐trained neural network for decryption. The PCC, MSE, SSIM, and PSNR between the decrypted and original images are marked under the corresponding decrypted images. c) Loss function during training and evaluation. The inset shows the learning rate during network training. d) The average PCC between the decrypted and original plaintexts during network training and evaluation. b) Plaintext image I: Reproduced under terms of the CC‐BY 2.0 license. Copyright 2015, Lawrence Lessig at Second Home London, by Innotech Summit, Flickr (https://www.flickr.com/photos/115363358@N03/18260388752/). P laintext image II: Reproduced under terms of the Public Domain Mark 1.0 license. Copyright 2018, kỉ yếu 12c, by khanhkhokhao201, Flickr (https://www.flickr.com/photos/154663983@N08/28538465128/). P laintext image III: Reproduced under terms of the Public Domain Mark 1.0 license. Copyright 2016, Future Leaders of the Pacific 2016 by US Embassy, Flickr (https://www.flickr.com/photos/us_embassy_newzealand/29355772191/). Plaintext image IV: Reproduced under terms of the CC‐BY 2.0 license. Copyright 2018, Ekaterina by Wonder Woman, Flickr (https://www.flickr.com/photos/zamerzla/28685633938/). The original images were cropped and converted to gray‐scale.

For information decryption from speckles, a neural network is constructed first. The structure of the neural network is shown in **Figure** **3**a, which is a U‐Net^[^ ^28^ ^]^ concatenated with a complex fully connected layer^[^ ^21^ ^]^ and a normalization layer, and the dimension of the filters in each layer is denoted in a format of length × height × amount (see Experimental Section for details). Then the neural network is trained with 19 800 pairs of face images and their corresponding speckles (see Experimental Section for details). The loss function used for training the neural network is:

(1) $$\text{loss}\;\text{function}=\mathrm{MSE}\left(\hat{y},y\right)-\mathrm{PCC}\left(\hat{y},y\right)$$

where *y* is the ground truth and $\hat{y}$ is the predicted output from the neural network. Here, we adopt PCC to measure the overall similarity and mean square error (MSE) to measure the pixel‐wise error. The experimental results of the neural network are shown in Figure 3b–d. During the network training and evaluation, PCC gradually increases (Figure 3d) and MSE gradually decreases (Figure S1a, Supporting Information), indicating increasing similarity between the decrypted images and the original plaintext. Especially, PCC becomes greater than 0.9 after 30 training epochs, suggesting high fidelity in decryption. In addition, we also measure other commonly used criteria, including the structural similarity index measure (SSIM) and the peak signal to noise ratio (PSNR), defined as Equations (2)–(5) in Experimental Section. In Figure 3b, four groups of exampled plaintexts, ciphertexts, and decrypted images during network testing are shown. The PCC, MSE, SSIM, and PSNR between the decrypted images and the original plaintexts are marked under the decrypted images. Overall, the average PCC, MSE, SSIM, and PSNR among all testing data (not included in network training) are 0.9422, 0.0083, 0.6884, and 21.25, respectively, demonstrating high accuracy of information decryption, which is essential for face recognition in the next stage. After network training, the plaintexts can be deleted from the cryptosystem to avoid privacy data leakage.

**Figure 4:**
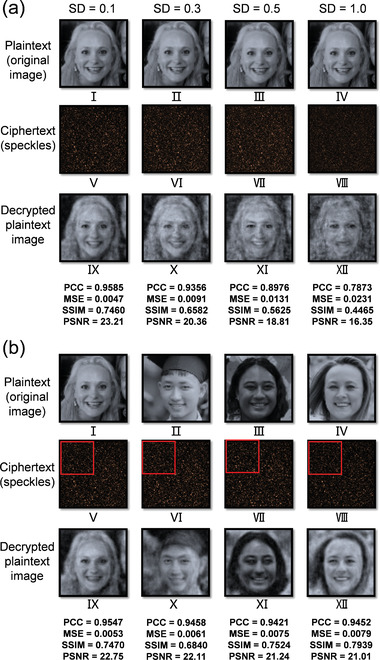
a) Decryption performance with noisy speckles: the speckles with computer‐generated random noise are fed into the pre‐trained neural network for decryption. The noisy speckles and the corresponding decrypted images are marked with the corresponding noise standard deviation (SD) and similarity criteria. b) Decryption performance with partial speckle patterns: only the top left corners (i.e., quarter field of view, marked in red box) of the speckle patterns are used to train, evaluate, and test the neural network. a) The plaintext image I‐IV: Reproduced under terms of the CC‐BY 2.0 license. Copyright 2015, Lawrence Lessig at Second Home London, by Innotech Summit, Flickr (https://www.flickr.com/photos/115363358@N03/18260388752/). b) The plaintext image I: Reproduced under terms of the CC‐BY 2.0 license. Copyright 2015, Lawrence Lessig at Second Home London, by Innotech Summit, Flickr (https://www.flickr.com/photos/115363358@N03/18260388752/). Plaintext image II: Reproduced under terms of the Public Domain Mark 1.0 license. Copyright 2018, kỉ yếu 12c, by khanhkhokhao201, Flickr (https://www.flickr.com/photos/154663983@N08/28538465128/). Plaintext image III: Reproduced under terms of the Public Domain Mark 1.0 license. Copyright 2016, Future Leaders of the Pacific 2016 by US Embassy, Flickr (https://www.flickr.com/photos/us_embassy_newzealand/29355772191/). Plaintext image IV: Reproduced under terms of the CC‐BY 2.0 license. Copyright 2018, Ekaterina by Wonder Woman, Flickr (https://www.flickr.com/photos/zamerzla/28685633938/). The original images were cropped and converted to gray‐scale.

Besides, the noise‐resisting ability of the network is examined since noise always exists in experiments due to environmental disturbances, vibration, airflow, et al.^[^ ^22^ ^]^ In our study, some computer‐generated Gaussian noise with different standard deviations (i.e., different noise amplitudes)^[^ ^29^ ^]^ is added to the speckles for testing, and the decryption performance is updated with the pre‐trained neural network. The results are given in **Figure** **4**a and Table S1, Supporting Information. In Table S1, Supporting Information, the PCCs are all greater than 0.9 when the standard deviations of the noise are ≤0.3, which is consistent with what can be seen in Figure 4a. The quality of the decrypted images deteriorates considerably when the standard deviation of the noise is ≥0.5 (i.e., noise amplitude is half of the mean of the signal amplitude), and the face outline becomes indistinct. These results suggest that the neural network trained in this study can handle low and moderate noise conditions to the testing data, which is meaningful to the applicability of the method.

Furthermore, due to multiple light scattering and the conceptualized infinite information channels^[^ ^16^ ^]^ within the scattering medium, it is hypothesized that the information in the plaintext is scrambled and distributes to the whole field of view (FOV) of the speckle pattern. Spatially, this speckle pattern could be large in practice, especially if the incident light is focused onto the front sample surface or the detection plane is far away from the sample. It is thus possible that only part of the speckle pattern is captured by the detection camera in experiments.^[^ ^30^ ^]^ To study whether this factor may affect the performance, an additional group of experiments is conducted by using a quarter FOV of the speckle patterns for network training, evaluation, and testing. That is, the dimension of the speckle patterns is reduced from 256 × 256 to 128 × 128 under the same spatial sampling condition. The experimental results are shown in Figure 4b, and Tables S2 and S3, Supporting Information. As seen, partial FOV leads to decryption results (Figure 4b) that are very comparable to those obtained with larger FOV (Figure 3b), confirming the hypothesis above. Such a non‐point‐to‐point information mapping between the plaintext and the ciphertext is distinctive to most existing cryptosystems. It allows smaller speckle FOVs to be adopted in network training, evaluation, and testing, which can relieve the burdens of data collection, storage, and processing without compromising the decryption accuracy.

### 2.3 Face Recognition

**Figure 5:**
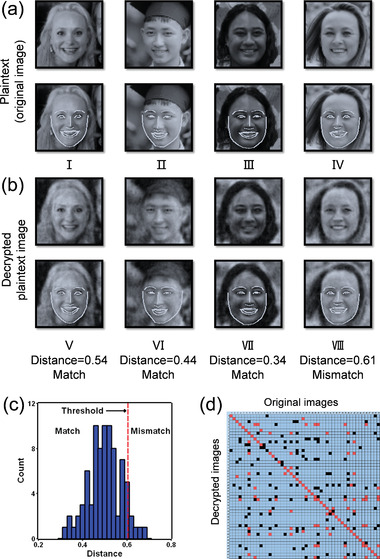
Face recognition results based on face images from FFHQ and the corresponding decrypted images from speckles. a) The original face images (i.e., plaintext) and their key features for face recognition. b) The decrypted face images by feeding speckle patterns into the trained neural network and their key features. The face encoding distances between the decrypted and original face images with a threshold = 0.6 are marked under the decrypted images. c) Face encoding distances between the decrypted and original images in the test dataset. If the distance is less than or equal to the threshold = 0.6, the recognition result is “Match”; otherwise, it is “Mismatch.” d) The face recognition results of the decrypted images. True positives are marked in red, true negatives are marked in blue, while false positives and false negatives are marked in black. a) First‐row plaintext image I: Reproduced under terms of the CC‐BY 2.0 license. Copyright 2015, Lawrence Lessig at Second Home London, by Innotech Summit, Flickr (https://www.flickr.com/photos/115363358@N03/18260388752/). The first‐row plaintext image II: Reproduced under terms of the Public Domain Mark 1.0 license. Copyright 2018, kỉ yếu 12c, by khanhkhokhao201, Flickr (https://www.flickr.com/photos/154663983@N08/28538465128/). First‐row plaintext image III: Reproduced under terms of the Public Domain Mark 1.0 license. Copyright 2016, Future Leaders of the Pacific 2016 by US Embassy, Flickr (https://www.flickr.com/photos/us_embassy_newzealand/29355772191/). First‐row plaintext image IV: Reproduced under terms of the CC‐BY 2.0 license. Copyright 2018, Ekaterina by Wonder Woman, Flickr (https://www.flickr.com/photos/zamerzla/28685633938/). The original images were cropped and converted to gray‐scale.

During decryption, we utilize PCC and other criteria to test similarities. However, these criteria are not suitable for face recognition as they may be affected by many factors other than face features, such as backgrounds, orientations, and expressions of faces.^[^ ^31^ ^]^ Therefore, at this stage the original and decrypted face images are further processed with an open‐source Python face‐recognition library.^[^ ^32^ ^]^ The neural network used for face recognition is based on ResNet,^[^ ^33^ ^]^ which is well‐trained based on 3 million faces, with 99.38% accuracy on the Labeled Faces in the Wild benchmark.^[^ ^34^, ^35^ ^]^ The face recognition network encodes each face image with a unique 128‐dimensional vector, which extracts the specific features of human faces, including eyebrows, eyes, noses, mouths, and cheeks. If the Euclidean distance^[^ ^36^ ^]^ between two face vectors is lower than a pre‐set threshold, two corresponding faces are defined as “Match” with each other; otherwise, they are defined as “Mismatch”, as exampled in **Figure** **5**c. The commonly used pre‐set threshold is 0.6 (for general situations) or 0.5 (for higher security scenes).

In our study, various thresholds between 0.5 and 0.6 are tested with decrypted face images illustrated in Figure 3. As an example, the results of face recognition with a threshold distance of 0.6 are shown in Figure 5. The key features of the original and decrypted face images from Figure 3b are extracted by the face recognition neural network and marked in the second row of Figures 5a and 5b, respectively.^[^ ^32^ ^]^ As seen, most of these decrypted images appear akin to their corresponding original plaintext images (e.g., image pairs I–V, II–VI, and III–VII, whose PCC are all more than 0.94) and hence are recognized as “Match.” Note that, however, some image pairs seem visually alike, such as IV–VIII whose PCC ≈ 0.96, but are still recognized as “Mismatch” since the Euclidean distance is 0.61, being above the threshold of 0.6. Nevertheless, it shows that the face recognition library can extract key features and scale the differences between the decrypted and original face images.

Furthermore, we test the accuracy of face recognition. The 128‐dimension face encodings from the decrypted images are compared with the corresponding encodings from the original face images, as shown in Figure 5d. The results with different distance thresholds are shown in **Table** **1** and compared with other face recognition algorithms.^[^ ^37^, ^38^, ^39^, ^40^, ^41^ ^]^ It is not surprising that different thresholds result in different recalls, precisions, and accuracies (Equations (9)–(12) in Experimental Section). It can be observed that our accuracy reaches greater than 98% when the threshold is below 0.58. Compared with FaceNet and VGGFace, the method proposed in this work has higher accuracy and is therefore more suitable for practical applications.^[^ ^38^, ^39^ ^]^ Moreover, the precision is 100% when the threshold is set at 0.5, indicating high confidence during face recognition. However, the recall and F1 score obtained in this study are not as good as those from FaceNet and VGGFace, which can be attributed to the fact that there are more negative samples than positive samples in the data we use. The performance can be further improved by adjusting the threshold in face recognition according to the sample distribution in the dataset, or tuning the structure or parameters of the neural network.

**Table 1:** Face recognition results by our method and other algorithms with optimal thresholds

	Threshold	Recall	Precision	Accuracy	F1 score
This work	0.60	66.18%	64.02%	97.87%	65.08%
	0.58	62.73%	69.66%	98.49%	66.01%
	0.56	61.65%	78.10%	98.93%	68.91%
	0.54	61.34%	87.95%	99.19%	72.28%
	0.52	56.07%	92.31%	99.25%	69.77%
	0.50	46.53%	100.00%	99.22%	63.51%
FaceNet^[^ ^38^ ^]^	0.90	96.42%	100.00%	98.21%	98.18%
VGGFace^[^ ^39^ ^]^	0.79	80.71%	97.41%	89.28%	88.28%
OpenFace^[^ ^40^ ^]^	0.47	16.42%	95.83%	57.85%	28.04%
DeepFace^[^ ^41^ ^]^	0.51	9.28%	100.00%	54.64%	16.99%

## 3 Discussions

In this study, a speckle‐based optical cryptosystem is proposed, implemented, and demonstrated, by exploiting a ground glass scattering medium as the physical secret key to generate speckle patterns that uniquely encrypt information. As for a cryptosystem, security is the topmost concern, and we will discuss the security of the proposed method from three aspects.

### 3.1 Length of the Secret Key

The equivalent key length of the scattering medium can be modelled by the transmission matrix, whose dimension in this work is (256 × 256) × (64 × 64), and each element is 64 bits (for complex float numbers) in the computer. Thus, the digital key of this cryptosystem is of length 64 × [(256 × 256) × (64 × 64)] = 1.72 × 10^10^ bits, that is, 17.2 gigabits, which is enormous for brute force attacks even with a quantum computer. In comparison, for pure software‐based encryption approaches, such as Advanced Encryption Standard (AES)^[^ ^5^ ^]^ and Compression Friendly Encryption Scheme (CFES),^[^ ^42^ ^]^ the digital cryptosystems are all based on matrix manipulations. As the size of the matrix (i.e., digital secret key length) increases, more multiplicative manipulations are needed, and the computational complexity grows exponentially. Therefore, to balance the computational efficiency and security, the digital secret key lengths in digital cryptosystems are usually limited to hundreds of bits. However, in our speckle‐based physical encryption process, no mathematical algorithms are involved, so the computational burden can be ruled out during encryption and users can achieve high security without compromising encryption speed. Note that, when it comes to decryption, our optical cryptosystem involves a large amount of computation. Fortunately, these decryption processes can be accelerated by using a high‐performance graphics processing unit (GPU).

### 3.2 Unclonable Feature of the Secret Key

**Figure 6:**
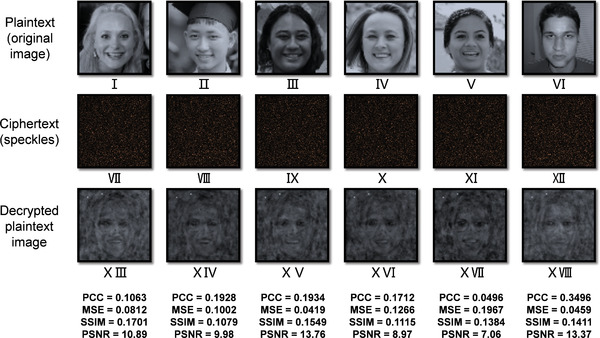
Wrong physical secret key attack: the same plaintext images are used, but another scattering medium is utilized to generate the speckles (i.e., ciphertext), which are input to the pre‐trained neural network to yield the decrypted plaintext images. The PCC, MSE, SSIM, and PSNR between the decrypted and the corresponding original face images are marked. The transmission matrix similarity, as measured by PCC, between the correct and wrong physical secret keys is 0.00012. Plaintext image I: Reproduced under terms of the CC‐BY 2.0 license. Copyright 2015, Lawrence Lessig at Second Home London, by Innotech Summit, Flickr (https://www.flickr.com/photos/115363358@N03/18260388752/). Plaintext image II: Reproduced under terms of the Public Domain Mark 1.0 license. Copyright 2018, kỉ yếu 12c, by khanhkhokhao201Flickr (https://www.flickr.com/photos/154663983@N08/28538465128/). Plaintext image III: Reproduced under terms of the Public Domain Mark 1.0 license. Copyright 2016, Future Leaders of the Pacific 2016 by US Embassy, Flickr (https://www.flickr.com/photos/us_embassy_newzealand/29355772191/). Plaintext image IV: Reproduced under terms of the CC‐BY 2.0 license. Copyright 2018, Ekaterina by Wonder Woman, Flickr (https://www.flickr.com/photos/zamerzla/28685633938/). Plaintext image V: Reproduced under terms of the Public Domain Mark 1.0 license. 2015, Resiliency Day, Sept. 11, Copyright 2015 by Presidio of Monterey, Flickr (https://www.flickr.com/photos/presidioofmonterey/21442846325/). Plaintext image VI: Reproduced under terms of the CC‐BY 2.0 license. Copyright 2008, P1020227 by Kyle Peyton, Copyright 2008, Flickr (https://www.flickr.com/photos/kylepeyton/2779218214/). The original images were cropped and converted to gray‐scale.

As for the optical setup, it is nearly impossible to generate the same speckles with a different scattering medium (i.e., the physical secret key), in which the scatterers are randomly distributed. The light–medium interactions are very complicated, and the resultant optical propagation involves intricate multipath scattering; minor variations in the scattering medium can influence the optical field considerably, resulting in a totally different transmission matrix of the scattering medium. Therefore, compared with existing digital encryption matrix‐based approaches (i.e., relays only on digital secret keys),^[^ ^43^ ^]^ it is nearly impossible to duplicate the inhomogeneous refractive index distribution of the scattering medium to crack the cryptosystem, except for a self‐defined medium such as a metasurface.^[^ ^44^, ^45^ ^]^ Therefore, the speckles can be viewed as nearly unclonable, and the decryption process is exclusive to the quantification of the scattering medium, that is, a deep neural network (DNN) trained with ciphertext (i.e., speckles) as the input and plaintext as the output. If speckles generated from another scattering medium (i.e., wrong physical secret keys) are input to the pre‐trained neural network for decryption, as exampled in **Figure** **6**, the decrypted results (XIII to XVIII) are obscure and very different from the plaintext (I to VI). Consequentially, the decrypted images cannot be used for face recognition, and thus the security of the proposed system can be guaranteed.

### 3.3 Uniqueness of the Optical Setup

Under extreme situations when hackers have obtained the scattering medium (i.e., the physical secret key), to produce the same speckle patterns, the error in duplicating the optical system alignment and the light–medium interaction should be within the optical wavelength scale.^[^ ^46^ ^]^ That is, the optical setup ensures that the interaction between the light and medium is hard to be reproduced due to the “narrow” range (approximately milliradians for tilt and submicron for shift) of the “memory effect.” What's more, within memory effect, neural networks can be built to retrieve images from speckle autocorrelations, and the trained neural networks can be generalized to unknown scattering media, that is, the trained neural networks based on speckle autocorrelations can be used for a ciphertext‐only attack. However, beyond the memory effect, it is theoretically impossible to build and train neural networks based on speckle autocorrelations to decrypt complex‐structured face images from an unknown scattering medium, due to weak relations between speckle autocorrelations and image autocorrelations.^[^ ^46^, ^47^ ^]^ In this work, the memory effect range is less than a quarter of the face image size, thus the cryptosystem is safe under ciphertext‐only attacks. Furthermore, chosen‐plaintext and known‐plaintext attacks are possible only when attackers can get access to at least 10 000 image‐speckle sets, as discussed in Figure S2, Supporting Information. In the proposed cryptosystem, obtaining such a large number of image‐speckle sets is possible only when attackers have access to the optical setup and the unique physical secret key simultaneously, which, however, is already beyond the scope of the topic. Even in that situation, if the unique physical secret key is stolen, it can be replaced with a new secret key to protect data.

### 3.4 Others

The intervention of optics further boosts the efficiency of encryption (i.e., at the speed of light), which overwhelms the software‐based cryptosystems. Optical solutions, including the proposed speckle‐based method and the DRPE method, can enable highly efficient encryption and generate high‐dimensional secret keys.^[^ ^8^ ^]^ Notably, compared with DRPE, the proposed method is advantageous due to its simpler optical design. DRPE requires two SLMs in the optical setup since the information is encrypted by two random phase masks.^[^ ^13^ ^]^ In our cryptosystem, the encryption can be performed with a scattering medium only. This not only facilitates the integration with other systems, but also reduces the cost of the cryptosystem. The most expensive component in the current system is the SLM, which is only responsible for loading the images and is indeed replaceable in practice since direct illumination of human faces can be used as input images for the cryptosystem. As a result, the cost of the proposed cryptosystems becomes comparable to the software‐based cryptosystems, which only require computers for encryption and decryption.

When it comes to system latency, although well‐known edge computing can help to recognize face images and protect privacy through computing in cloudlets, its scalability is refrained by the computing power, leading to applications of limited database.^[^ ^48^ ^]^ In comparison, the proposed light‐based system can achieve fast encryption speed and high scalability. Moreover, with the development of high throughput communication networks, such as 5G, the latency of the proposed system can also be comparable to edge computing‐based face recognition.^[^ ^49^ ^]^

When it comes to the quality of decrypted images, the proposed neural network delivers high similarity between decrypted and original images, resulting in accurate face recognition (i.e., 98%) that is comparable to other state‐of‐art methods.^[^ ^37^, ^38^, ^39^, ^40^, ^41^ ^]^ That said, some high‐frequency information (i.e., detailed structures, such as hair) in images may still be lost after the speckle‐based encryption in experiments, due to non‐ideal experimental setups such as aberrations from the SLM curvature, optical lens, and camera. The lost high‐frequency information is therefore difficult to be restored by neural networks during decryption. Furthermore, to simplify the optical setup, we have just recorded speckle intensity during experiments. The missing phase of the speckle field also results in information loss. These all lead to moderate PSNR of decrypted images, which will be improved in the next phase of study by optimizing the optical setup and/or the neural network structures.

Last but not least, let us highlight the novelty of the proposed speckle‐based optical cryptosystem from three aspects. First, although some literature has mentioned speckle‐based encryption recently,^[^ ^14^, ^15^ ^]^ they have mainly focused on the encryption of simple digits and characters, but not complex‐structured images such as face images in this work. The cryptosystem for face reconstruction and recognition is considerably more complicated than that for digits and characters. Second, although learning‐based decryption has also been demonstrated,^[^ ^14^, ^19^ ^]^ our efforts have gone beyond. After decryption with high fidelity, face recognition is demonstrated with 98% accuracy, which is comparable to the state‐of‐art algorithms in the field. Third and most importantly, the proposed speckle‐based optical cryptosystem has a very high level of security. The length of the physical security key is more than 17 gigabits, being many magnitudes longer than that of pure software‐based encryption approaches and sufficiently secure for brute force attacks. Due to the nature of the speckle‐based mechanism, there is no computational burden or compromised speed during encryption. Meanwhile, the complicated light–medium interaction assures every physical secret key (i.e., the scattering medium) is unique and nearly unclonable. Furthermore, the narrow memory effect range of the optical system determines that the interaction between the light and the medium is hard to be reproduced, keeping the cryptosystem from ciphertext‐only attacks, chosen‐plaintext attacks, and known‐plaintext attacks. The only exception is when the optical setup and the physical secret key are both leaked, which, however, is beyond what a cryptosystem can handle.

## 4 Conclusion

In this work, we demonstrate a speckle‐based optical cryptosystem for face recognition, and the accuracy in this study has reached more than 98%, which is comparable to that of other state‐of‐art methods. With the proposed speckle‐based optical cryptosystem, the encrypted private data (e.g., face images) is difficult to crack and reduces the risk of information leakage. The speckle‐based optical cryptosystem is suitable for practical applications due to its high security, fast speed, low cost, insensitivity to the FOV, as well as immunity to low and moderate noise to the ciphertexts. That said, the accuracy of face recognition can still be further improved by constructing more complex neural networks that lead to an all‐speckle‐based optical cryptosystem for decryption and face recognition,^[^ ^50^, ^51^ ^]^ where there is no need to decrypt optical speckles to face images. Moreover, to further enhance the security of the encryption processes, multi‐channel laser diffraction by high‐dimensional scattering media can be adopted to increase the speckle randomness. On the other hand, binary speckles can be used to reduce data storage space and increase data transmission speed.^[^ ^52^ ^]^ Collectively, although this study contains only a proof‐of‐principle demonstration for face encryption and recognition, we believe that with further optimization, the proposed speckle‐based optical cryptosystem may find or inspire wide applications in high‐security information encryption and decryption.

## 5 Experimental Section

### Optical Setup

The experimental setup during speckle encryption is shown in Figure 2. First, the human face images were loaded onto the SLM (HOLOEYE PLUTO VIS056 1080p, German). The human face images used here were taken from the thumbnails of FFHQ database, a dataset of human face images.^[^ ^27^ ^]^ The original FFHQ database contains 70 000 images, from which the first 20 000 images were selected for demonstrations in this study. Light from a continuous wave 532 nm laser (EXLSR‐532‐300‐CDRH, Spectra‐Physics, Excelsior Scientific Continuous‐wave laser, Single mode, 300 mW, USA) was expanded by a 4‐f system (L1 and L2 in Figure 2) so that the SLM was fully illuminated to modulate the incident light. In experiments, the resolution of the SLM was 1920 × 1080, and the 128 × 128 thumbnails were up‐sampled (8‐time nearest interpolation) to 1024 × 1024 and loaded onto the SLM to fully utilize the modulation capability. The grey‐scale intensity of the image (distributed from 0 to 255) on the SLM was then converted to a phase delay (0 to 2*π*). Finally, the wavefront‐modulated beam light was focused by an objective lens (RMS20X, Olympus, Japan) onto and propagated through a scattering medium (220‐grid ground glass, diameter of 1.0 inch, DG10‐220‐MD, Thorlabs, USA). In these experiments, 20 000 images were sequentially loaded onto the SLM, and the corresponding speckle patterns were captured by a CMOS camera (FL3‐U3‐32S2M‐CS, PointGrey, Canada) with a resolution of 256 × 256.

### Training Dataset

The speckles used as the network input were 256 × 256 speckle images captured by the CMOS camera, and the images used as the network output were 64 × 64 images that were down‐sampled through two‐pixel‐binning from the FFHQ dataset (128 × 128) to avoid using up the GPU memory.^[^ ^27^ ^]^ These resolutions were chosen to make full use of the experimental setup and achieve high fidelity image decryption, as discussed in Figure S3, Supporting Information. The amount of the whole dataset was 20 000: 19 800 image‐speckle pairs for training, 100 image‐speckle pairs for testing, and 100 image‐speckle pairs for evaluation. Before the speckle data was input to the neural network, the input data were linearly normalized to [0,1] for better neural network performance.^[^ ^53^ ^]^

### Neural Network for Decryption

The detailed structure of the neural network for decryption is shown in Figure 3a. Overall, the architecture of the neural network was based on the commonly used U‐Net^[^ ^28^ ^]^ with an additional complex fully connected layer^[^ ^21^ ^]^ and a normalization layer. The encoders in the U‐Net contained 4 down‐sampling blocks and the decoders in the U‐Net contained 4 up‐sampling blocks. In addition, the fully connected layer was based on complex numbers. In Figure 3a, the blue arrows and filters represented the encoders in the U‐Net, and the orange arrows and filters represented the decoders in the U‐Net. The encoder tended to extract low‐dimensional features from the speckles and encoded them, and the decoder then tended to extract high‐dimensional features and decoded them.^[^ ^28^ ^]^ As a result, the encoder and decoder‐shaped neural network could extract features of different dimensions. The fully connected layer was used as the last layer to transform extracted features into images. The normalization layer limited the output range to [0,1]. At last, the final output was the face images decrypted from random speckles, which were then used for face recognition. During neural network training, the optimizer used in training the neural network was stochastic gradient descent (SGD),^[^ ^54^ ^]^ and the learning rate was 0.15, with cosine annealing. During the experiments, the neural network was trained for 30 epochs, and the neural network was then tested. The software framework used was Pytorch 1.8.0 with Python 3.7.6 and Compute Unified Device Architecture (CUDA) 10.1 for GPU acceleration. The hardware used was Dell Precision Tower 5810 with Intel Xeon E5‐1650 V3 CPU, 64 GB RAM, and Nvidia GeForce RTX 2080Ti 11GB GPU. During the training, one epoch took ≈30 min, and the whole training process takes ≈15 h.

### Image Similarity Criteria

During neural network training, evaluation, and testing, PCC, MSE, PSNR, and SSIM were used as the image similarity criteria, which are defined in Equations (2)–(5):

(2) $$\mathrm{PCC}=\frac{\mathrm{mean}\left[\left(y-\mathrm{mean}\left(y\right)\right)\times \left(\hat{y}-\mathrm{mean}\left(\hat{y}\right)\right)\right]}{\mathrm{std}\left(y\right)\times \mathrm{std}\left(\hat{y}\right)}$$

(3) $$\mathrm{MSE}=\mathrm{mean}[{\left(\hat{y}-y\right)}^{2}]$$

(4) $$\mathrm{PSNR}=20\times \log_{10}\frac{\max\left(\hat{y},y\right)}{\sqrt{\mathrm{MSE}}}$$

(5) $$\mathrm{SSIM}=l\left(\hat{y},y\right)\times c\left(\hat{y},y\right)\times s\left(\hat{y},y\right)$$

(6) $$l\left(\hat{y},y\right)=\frac{2\times \mathrm{mean}\left(y\right)\times \mathrm{mean}\left(\hat{y}\right)+c_{1}}{\mathrm{mean}{\left(y\right)}^{2}+\mathrm{mean}{\left(\hat{y}\right)}^{2}+c_{1}}$$

(7) $$c\left(\hat{y},y\right)=\frac{2\times \mathrm{std}\left(y\right)\times \mathrm{std}\left(\hat{y}\right)+c_{2}}{\mathrm{std}{\left(y\right)}^{2}+\mathrm{std}{\left(\hat{y}\right)}^{2}+c_{2}}$$

(8) $$s\left(\hat{y},y\right)=\frac{\mathrm{cov}\left(y,\hat{y}\right)+c_{3}}{\mathrm{std}\left(y\right)\times \mathrm{std}\left(\hat{y}\right)+c_{3}}$$

In the equations above, *y* and $\hat{y}$ are the original and decrypted images, respectively; *mean*(*y*) and $\mathrm{mean}(\hat{y})$ are the mean values of *y* and $\hat{y}$, respectively; *std*(*y*) and $\mathrm{std}(\hat{y})$ are the standard deviation of *y* and $\hat{y}$, respectively; $cov(y,\hat{y})$ is the covariance of *y* and $\hat{y}$; *c*_1_, *c*_2_, and *c*_3_ are three very small constants (10^−5^) to prevent division by 0 in SSIM;^[^ ^55^ ^]^ $l(\hat{y},y)$ is the luminance similarity; $c(\hat{y},y)$ is the contrast similarity; $s(\hat{y},y)$ is the structure similarity. Among these criteria, only MSE and PCC were used in the loss function during network training, and the other criteria were just used during network evaluation and testing.

### Face Recognition Criteria

The decrypted images were input to an open‐source face recognition program for face recognition.^[^ ^32^ ^]^ Before testing the neural network, some images with sunglasses and babies were excluded since some of their facial key points were ambiguous. The most important criterion during network testing was face recognition accuracy. First, the face recognition program encodes each face image's specific features (including eyebrows, eyes, noses, mouths, and cheeks, as illustrated in Figure 5a,b) with one special 128‐dimension encoding,^[^ ^32^ ^]^ which took less than 1 s. Then, the target is that if the Euclidean distances^[^ ^36^ ^]^ between the encoding vectors of two original images are smaller than the preset threshold (indicating that they are the same person), the distances between the two corresponding decrypted images are also expected to be smaller than the preset threshold, indicating that the people in the decrypted images and the original images are “match.” Here, mainstream computers to date (e.g., Xeon E5‐1650 V3 with 6 cores in experiments) could handle more than 10 000 face encoding distances within 1 s. The encodings of the decrypted images were also compared with each encoding of the original images. If the two original images’ encoding distances were smaller than the preset threshold, the two samples were treated as positive samples. And if the corresponding two decrypted images’ encoding distances are also smaller than the preset threshold, the results are true positives, otherwise, they are false negatives. On the contrary, if the two original images’ encoding distances are larger than the preset threshold, the two samples are treated as negative samples. And if the corresponding two decrypted images’ encoding distances are also larger than the preset threshold, the results are true negatives; otherwise, they are false positives. During network testing, precision, recall, F1‐score, and accuracy were used to test the performance, as defined in Equations (9)–(12).

(9) $$\mathrm{Recall}=\frac{\text{True}\;\text{Positive}}{\text{True}\;\text{Positive}+\text{False}\;\text{Negative}}$$

(10) $$\mathrm{Precision}=\frac{\text{True}\;\text{Positive}}{\text{True}\;\text{Positive}+\text{False}\;\text{Positive}}$$

(11) $$\begin{array}{c} \begin{array}{c} \mathrm{Accuracy}=\frac{\text{True}\;\text{Positive}+\text{True}\;\text{Negative}}{\text{True}\;\text{Negative}+\text{True}\;\text{Positive}+\text{False}\;\text{Negative}+\text{False}\;\text{Positive}} \end{array} \end{array}$$

(12) $$F1\text{score}=2\times \frac{\text{Precision}\times \text{Recall}}{\mathrm{Precision}+\mathrm{Recall}}$$

As one person might be recognized as two different people, while two different people should not be recognized as the same person, accuracy is more meaningful than the other three criteria in this study.

## Conflict of Interest

The authors declare no conflict of interest.

## Author Contributions

Q.Z., H.L., and Z.Y. contributed equally to this work. Q.Z., H.L., Z.Y., J.T., and P.L. conceived the idea. Q.Z., H.L., and Z.Y. conducted the experiments and processed the data. Q.Z., H.L., Z.Y., and C.M.W. wrote the manuscript. J.T. and P.L. supervised the project. All members contributed to the discussion of the results and proofreading of the manuscript.

## Supporting information

Supporting Information

## Data Availability

The data that support the findings of this study are available from the corresponding author upon reasonable request.
