# Supplementary material for: Speckle‐Based Optical Cryptosystem and its Application for Human Face Recognition via Deep Learning
Source: Adv Sci (Weinh). 2022 Jun 24;9(25):2202407. doi: 10.1002/advs.202202407 (PMC9443436; doi:10.1002/advs.202202407)
Supplement: Supplementary file 1 — Supporting Information [file ADVS-9-2202407-s001.pdf]

ADVANCED SCIENCE
Open Access

Supporting Information

for *Adv. Sci.*, DOI 10.1002/advs.202202407

Speckle-Based Optical Cryptosystem and its Application for Human Face Recognition via Deep Learning

Qi Zhao, Huanhao Li, Zhipeng Yu, Chi Man Woo, Tianting Zhong, Shengfu Cheng, Yuanjin Zheng, Honglin Liu, Jie Tian and Puxiang Lai**

Supporting Information

Speckle-based Optical Cryptosystem and its Application for Human Face Recognition via Deep Learning

Qi Zhao,† Huanhao Li,† Zhipeng Yu,† Chi Man Woo, Tianting Zhong, Shengfu Cheng, Yuanjin Zheng, Honglin Liu, Jie Tian, and Puxiang Lai**

1. Neural network training and evaluation results

We trained the neural network for 30 epochs, during which the training and evaluating loss functions gradually decrease with epochs, and the learning rate anneals like a cosine function to avoid overfitting. The MSE, PSNR, and SSIM during training are shown in **Figure S1**.

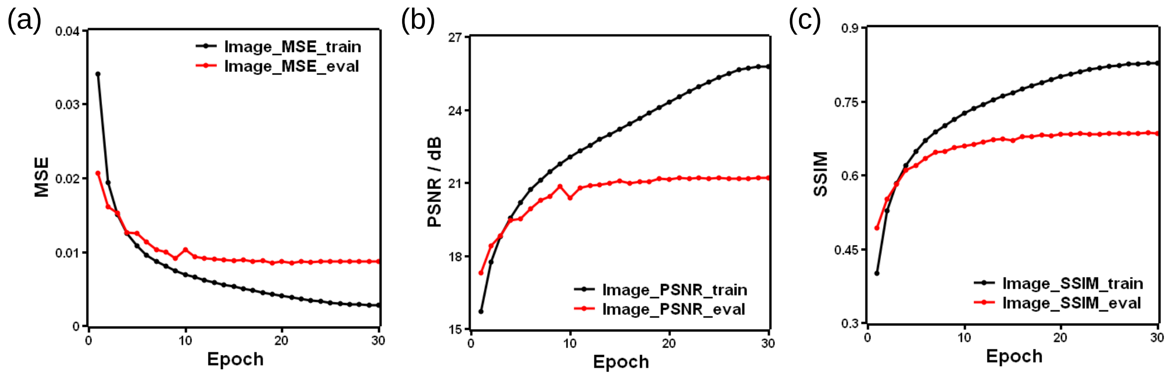

Figure S1. The similarity criteria between the decrypted and original images during network training and evaluation: (a) MSE, (b) PSNR, (c) SSIM.

2. Neural network testing with noisy speckles

To evaluate the influence of noise on image decryption, we added some noise to the speckles and tested the pre-trained neural network. The noise was computer-generated Gaussian noise with different standard deviations (i.e., different noise amplitudes). The numerical results are shown in Table s1, and the visual results are shown in Figure 4a. As seen in Table s1, PCC is greater than 0.9 when the standard deviation of the noise is ≤ 0.3. And SSIM is greater than 0.6, PSNR is greater than 20 [dB], and MSE is smaller than 0.1, when the standard deviation is ≤ 0.2. In Figure 4a, the decrypted images appear worse when the standard deviation of the noise is ≥ 0.5 (i.e., noise amplitude is half of the mean of the signal amplitude), and the face outlines are indistinct. As for face recognition, recalls and precisions are strongly affected by noise. The reason is that small differences between the decrypted and original images can lead

to "Mismatch". These results suggest that the pre-trained neural network can handle low and moderate noise conditions reasonably, which is meaningful to the applicability of the method.

Table S1. Network testing and face recognition results with noisy speckles

Noise standard deviations	PCC	MSE	SSIM	PSNR [dB]	Face	Noise standard deviations	PCC	MSE	SSIM
0	0.9422	0.0083	0.6884	21.25	0.60	66.18%	64.02%	97.87%	65.08%
					0.54	61.34%	87.95%	99.19%	72.28%
					0.50	46.53%	100.00%	99.22%	63.51%
0.1	0.9382	0.0086	0.6727	21.07	0.60	62.36%	52.00%	96.25%	56.71%
					0.54	60.15%	74.77%	98.84%	66.67%
					0.50	56.31%	95.08%	99.30%	70.73%
0.2	0.9285	0.0096	0.6384	20.54	0.60	60.52%	47.81%	95.85%	53.42%
					0.54	52.63%	73.68%	98.72%	61.40%
					0.50	45.63%	92.16%	99.13%	61.04%
0.3	0.9136	0.0118	0.5937	19.62	0.60	49.45%	39.88%	95.08%	44.15%
					0.54	42.86%	67.06%	98.49%	52.29%
					0.50	28.16%	87.88%	98.87%	42.65%
0.4	0.8967	0.0139	0.5535	18.85	0.60	52.40%	36.88%	94.60%	43.29%
					0.54	36.84%	54.44%	98.19%	43.95%
					0.50	20.39%	80.77%	98.74%	32.56%

3. Partial FOV used for image decryption

The field of view (FOV) of the speckles was shrunk from 256×256 to 128×128 for decryption, with results shown in Figure 4b and Table S2 & S3. We divided speckle patterns (256×256, full FOV) into four small FOVs (128×128), including top left, top right, bottom left, and bottom right. Then, we used the top left of the speckles to train and test the neural network. The neural network structure used here was the same as that of the neural network in Figure 2, except that the filter sizes were changed according to the FOV change. The visual features in Figure 4b appear alike to those in Figure 3b, except that the similarity criteria are different. This indicates that the neural network can still decrypt images with speckles of partial FOV, although the PCC of the decrypted images from 128×128 speckles is slightly lower than that

with 256×256 speckles. The numerical results in Table s2 are also comparable to those in Table 1.

Table S2. Face recognition results using the top left of speckles. The network structure used here is identical to that in Figure 2, except that the filter shapes are adjusted according to the FOV change.

Similarity criteria	Threshold	Recall	Precision	Accuracy	F1 score
MSE = 0.0095	0.60	57.93%	44.48%	95.50%	50.32%
PCC = 0.9339	0.58	56.72%	55.61%	97.42%	56.16%
PSNR = 20.72 [dB]	0.56	51.48%	66.41%	98.17%	58.00%
SSIM = 0.6603	0.54	53.38%	75.53%	98.77%	62.56%
	0.52	49.59%	84.72%	98.94%	62.56%
	0.50	43.69%	93.75%	99.11%	59.60%

Table S3. Neural network testing results using different small FOVs of speckles.

Field of view	MSE	PCC	SSIM	PSNR [dB]
Whole pattern	0.0083	0.9422	0.6884	21.25
Top left	0.0095	0.9339	0.6603	20.72
Top right	0.0094	0.9326	0.6518	20.72
Bottom left	0.0092	0.9347	0.6566	20.84
Bottom right	0.0095	0.9330	0.6528	20.69

A second group of experiments were performed with different FOVs of the speckles being used, and the results are shown in Table S3. As seen, the similarity criteria are rather consistent among different small FOVs, which again confirms the hypothesis that the wavefront information is distributed over the entire speckle field of view. Therefore, one can use small FOVs of speckles for decryption with reasonably compromised performance.

4. Experiment with different training dataset sizes

Chosen-plaintext attacks and known-plaintext attacks are possible only when attackers can simultaneously access the architecture of the neural network as well as the trained weights embedded in the neural network, which are generated from a large number of image-speckle sets. In **Figure S2**, experimental results with different training dataset sizes are shown, and

the testing datasets were identical to previous experiments. As seen, to achieve satisfactory performance, for example, PCC and face recognition accuracy be greater than 0.9, the training datasets need to be larger than 10,000 and 15,000, respectively. In the proposed cryptosystem, obtaining such a large number of image-speckle sets is possible only when attackers have access to the optical setup and the unique physical secret key simultaneously, which, however, is very demanding and already beyond the scope of the topic.

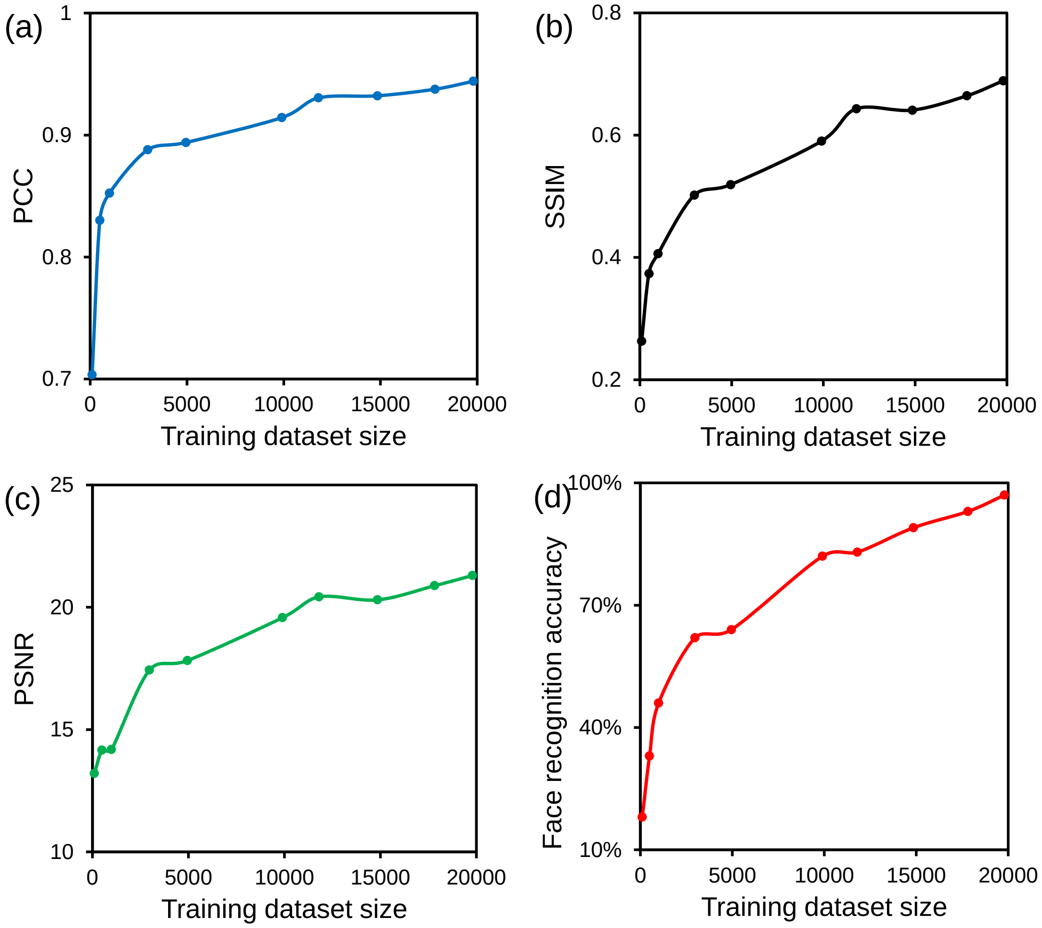

Figure S2. Experimental results with different dataset sizes: similarities between the decrypted and original images as measured by (a) PCC, (b) SSIM, and (c) PSNR, as well as (d) face recognition accuracy, as a function of training dataset size.

5. Experiment with different image resolutions

In this section, we compare experimental results with different image resolutions. The neural networks were trained and tested with output images of different resolutions. Since our GPU memory cannot handle neural networks with 256×256 speckle input and 128×128 image output, the resolution of input speckles was adjusted to 128×128 to reduce the GPU memory usage, i.e., input speckles were cropped from the top left corners of the whole 256×256 speckles. And the output image (i.e., human face) resolutions included 128×128, 64×64, and

32×32. In **Figure S3**, images with 64×64 resolution yield the best decryption fidelity (except for SSIM, which is sensitive to detailed structures in high-resolution images) and the highest face recognition accuracy. Results with 128×128 image resolution obtain the lowest face recognition accuracy, due to insufficient information in 128×128 speckles to decrypt 128×128 face images with lots of detailed structures. After balancing the computational cost, memory usage, decryption fidelity, and face recognition accuracy, we choose the image resolution of 64×64 in this work.

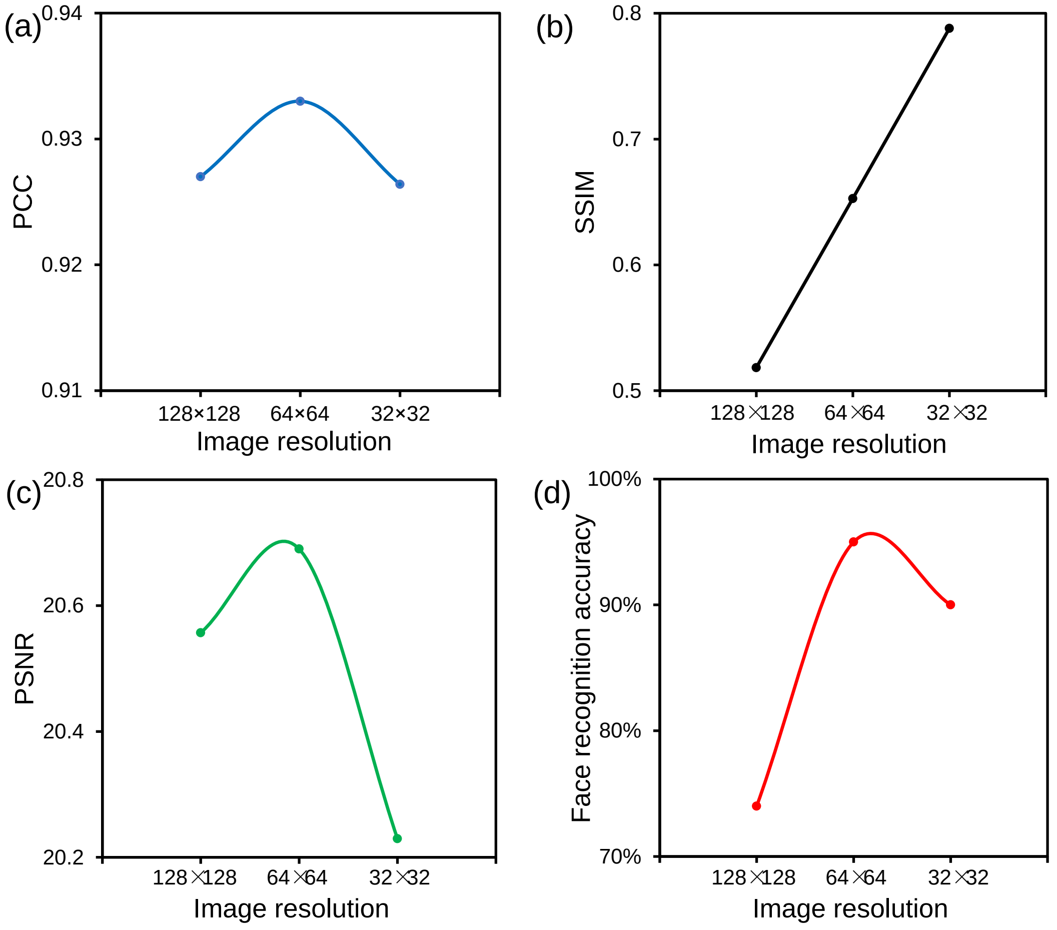

Figure S3. Experimental results with different image resolutions: similarities between the decrypted and original images as measured by (a) PCC, (b) SSIM, and (c) PSNR, as well as (d) face recognition accuracy, as a function of image resolution.

6. Code of the neural network for decryption

In this study, Python code, as attached below, was used to build the neural network to decrypt speckles. First, a four-layer U-Net was designed and constructed to extract features from speckles. The input and output of the U-Net were both 256×256. Then, the complex valued fully connected layer transferred the output of U-Net to images. And the normalization layer was utilized to normalize the output range to [0,1]. The size of the final output image was

64×64 to maximize the use of the GPU memory. During neural network building, the checkpoint technic was utilized to reduce the memory usage in building such a large-scale neural network model.

```
import torch
import torch.nn as nn

class _DenseLayer(nn.Module):
        # Submodule used in decoders and encoders
    def __init__(self, in_ch, out_ch, drop_rate=0.5):
        super(_DenseLayer, self).__init__()
        self.conv = nn.Sequential(
            nn.Conv2d(in_ch, out_ch, 3, 1, 1),
            nn.BatchNorm2d(out_ch),
            nn.ReLU(),
            nn.Dropout2d(drop_rate)
        )

    def forward(self, x):
        out = self.conv(x)
        return out

class DenseBlock(nn.Module):
        # Submodule used in decoders and encoders
    def __init__(self, in_ch, out_ch, growth_rate=16, num_layer=4, drop_rate=0.5):
        super(DenseBlock, self).__init__()
        for i in range(num_layer-1):
            layer = _DenseLayer(
                in_ch=in_ch + i*growth_rate,
                out_ch=growth_rate,
                drop_rate=drop_rate
            )
            self.add_module('denselayer%d' % (i+1), layer)
        self.out = nn.Sequential(
```

```
            nn.Conv2d(
                in_channels=in_ch + int(growth_rate*(num_layer-1)*(num_layer-2)/2),
                out_channels=out_ch,
                kernel_size=3,
                stride=1,
                padding=1),
            nn.BatchNorm2d(out_ch),
            nn.ReLU()
        )

    def forward(self, x):
        dimension = [x]
        for name, layer in self.named_children():
            new_dimension = layer(torch.cat(dimension, 1))
            dimension.append(new_dimension)
        #out = self.out(torch.cat(dimension, 1))
        return new_dimension

class down(nn.Module):
            # Encoders in UNet
    def __init__(self, dropout, in_ch, out_ch, kernel_size=4, padding=1, stride=2):
        super(down, self).__init__()
        self.same = nn.Sequential(
            DenseBlock(out_ch=out_ch, in_ch=in_ch, drop_rate=dropout),
            nn.Dropout2d(dropout)
        )
        self.d = nn.Sequential(
            nn.Conv2d(out_ch, out_ch, kernel_size=kernel_size, padding=padding, stride=stride),
            nn.BatchNorm2d(out_ch),
            nn.ReLU(),
            nn.Dropout2d(dropout)
        )

    def forward(self, x):
```

```
        x_skip = self.same(x)
        down = self.d(x_skip) if x.shape[-1]>1 else 0
        return x_skip, down

class up(nn.Module):
            # Decoders in UNet
    def __init__(self, dropout, in_ch, in_m_ch, out_ch, kernel_size=4, padding=1, stride=2):
        super(up, self).__init__()
        self.u = nn.Sequential(
            nn.ConvTranspose2d(in_ch, in_ch, kernel_size=kernel_size, padding=padding,
stride=stride),
            nn.BatchNorm2d(in_ch),
            nn.ReLU()
        )
        self.h = nn.Sequential(
            DenseBlock(out_ch=out_ch, in_ch=in_m_ch, drop_rate=dropout),
            nn.Dropout2d(dropout)
        )

    def forward(self, x, x_skip):
        x_up = self.u(x)
        x = torch.cat([x_up, x_skip], 1)
        x = self.h(x)
        return x

class top_out(nn.Module):
            # Top UNet output layer
    def __init__(self, dropout, in_ch, kernel_size=3, stride=1, padding=1):
        super(top_out, self).__init__()
        self.cnn = nn.Sequential(
            nn.Conv2d(in_ch, 128, kernel_size=kernel_size, stride=stride, padding=padding),
            nn.BatchNorm2d(128),
            nn.ReLU(),
            nn.Dropout2d(dropout),
```

```
            nn.Conv2d(128, 1, kernel_size=kernel_size, stride=stride, padding=padding),
            nn.Sigmoid(),
        )

    def forward(self, x):
        return self.cnn(x)

class unet(nn.Module):
            # UNet network construction
    def __init__(self, dropout=0, in_dim=256):
        super(unet, self).__init__()
        self.in_dim  = in_dim
        self.s1_down = down(dropout, in_ch=   1, out_ch=   64, kernel_size=4, padding=1,
stride=2)
        self.s2_down = down(dropout, in_ch= 64, out_ch= 128, kernel_size=4, padding=1,
stride=2)
        self.s3_down = down(dropout, in_ch=128, out_ch= 256, kernel_size=4, padding=1,
stride=2)
        self.s4_down = down(dropout, in_ch=256, out_ch= 512, kernel_size=4, padding=1,
stride=2)
        self.s5_down = down(dropout, in_ch=512, out_ch=1024, kernel_size=4, padding=1,
stride=2)
        self.s4_up = up(dropout, in_ch=1024, in_m_ch=1024+512, out_ch=256)
        self.s3_up = up(dropout, in_ch=256, in_m_ch=512, out_ch=128)
        self.s2_up = up(dropout, in_ch=128, in_m_ch=256, out_ch=64)
        self.s1_up = up(dropout, in_ch=64, in_m_ch=128, out_ch=32)
        self.output = top_out(dropout, in_ch=32)

    def forward(self, speckles):
        x_s1_skip, x_s1_down = self.s1_down(speckles.reshape(-1, 1, in_dim, in_dim))
        x_s2_skip, x_s2_down = self.s2_down(x_s1_down)
        x_s3_skip, x_s3_down = self.s3_down(x_s2_down)
        x_s4_skip, x_s4_down = self.s4_down(x_s3_down)
        x_bottom, _ = self.s5_down(x_s4_down) #16x16
```

```
        x_s4_up = self.s4_up(x_bottom, x_s4_skip)
        x_s3_up = self.s3_up(x_s4_up, x_s3_skip)
        x_s2_up = self.s2_up(x_s3_up, x_s2_skip)
        x_s1_up = self.s1_up(x_s2_up, x_s1_skip)
        out = self.output(x_s1_up)
        return out

def normalization(image): # Normalize the input to range [0,1]
    Min = image.min(-2,keepdims=True)[0].min(-1,keepdims=True)[0]
    Max = image.max(-2,keepdims=True)[0].max(-1,keepdims=True)[0]
    image = (image-Min)/(Max-Min)
    image = torch.nan_to_num(image)
    return image

class UNet_with_Complex_Full_Connect_Net(torch.nn.Module):
    def __init__(self,in_dim,out_dim,drop_out_rate=0):
        super(UNet_with_Complex_Full_Connect_Net,self).__init__()
        self.in_dim   = in_dim # Input speckle dimension
        self.out_dim  = out_dim # Output image dimension
        self.p        = drop_out_rate
        self.unet     = unet(drop_out_rate, in_dim=self.in_dim)
        self.FC       = torch.nn.Linear(in_dim*in_dim, out_dim*out_dim).to(torch.complex64)

    def forward(self,x):
            # UNet_with_complex_FC is made up of UNet and a complex number-based fully
connected layer
        x.requires_grad=True
        x = checkpoint(self.unet,x)
        x = x.reshape(-1, self.in_dim*self.in_dim).to(torch.complex64)
        x = checkpoint(self.FC,x)
            # Checkpoint is utilized to reduce the GPU memory use
        x = x.abs()
        x = x.reshape(-1, 1, self.out_dim, self.out_dim)
        x = normalization(x)
```

```
return x
```
